# HIV‐positive patients diagnosed with COVID‐19 in Central and Eastern European Countries

**DOI:** 10.1002/jcla.24675

**Published:** 2022-08-28

**Authors:** Agata Skrzat‐Klapaczyńska, Kerstin Kase, Justyna D. Kowalska

**Affiliations:** ^1^ Department of Adults' Infectious Diseases, Hospital for Infectious Diseases Medical University of Warsaw Warsaw Poland; ^2^ Tartu University Clinic Tartu Estonia

**Keywords:** Central and Eastern Europe, COVID‐19, HIV

To the Editor,

In their recent systematic review of case series and case reports about HIV‐positive patients diagnosed with COVID‐19, Heidary et al.^1^ underlined that HIV may increase the severity, morbidity, and mortality rates of COVID‐19 infection. In the opposite to these findings, among the first cases reported in early March 2020 in Central and Eastern Europe most HIV‐positive patients diagnosed with COVID‐19 were in good condition. Moreover, in other studies the course of COVID‐19 did not significantly different in patients with and without HIV infection.^2^, ^3^, ^4^, ^5^, ^6^, ^7^

In the beginning of the pandemic, there was concern about the course of COVID‐19 in HIV‐infected patients. Overall, it has been confirmed that people with other comorbidities, including cardiovascular disease, type II diabetes and chronic pulmonary diseases and older people are at higher risk of severe COVID‐19 in the general population.^8^ HIV‐infected individuals were considered to be at higher risk of COVID‐19 infection because significant proportion of them are over the age of 50, and the comorbidities such as chronic lung disease and cardiovascular disease are more common than in general population.^9^, ^10^

We provided retrospective analysis included all confirmed COVID‐19 cases between March 11 and June 26, 2020 among HIV‐positive patients in 12 countries (Estonia, Czech Republic, Lithuania, Albania, Belarus, Romania, Serbia, Bosnia and Herzegovina, Poland, Russia, Hungary, Bulgaria). We reported 34 COVID‐19 cases among HIV‐positive patients in total. HIV‐positive patients diagnosed with COVID‐19 characteristics included basic information (age, sex), information about HIV infection (nadir CD 4+ T‐cell count, baseline HIV RNA viral load [VL], time since HIV diagnosis, latest HIV VL prior COVID‐19 infection, most recent CD 4+ T‐cell count prior COVID‐19 infection), information about comorbidities and coinfections, information about cART regimen at the time of COVID‐19 infection, course of COVID‐19 infection (Table 1).

**TABLE 1:** HIV‐positive patients diagnosed with COVID‐19 characteristics

Characteristic	All (*n* = 34)	%
Age, median (IQR), year	42.7 (IQR = 35.8–48.5)	–
Male, sex	24	70.6
BMI, median (IQR), kg	25.2 (IQR = 20–30.1)	–
Time since HIV diagnosis in years	0.1–22	–
Nadir CD4 T‐cell count (IQR), cells/mm^3^	290 (IQR = 15–880)	–
Latest CD4 T‐cell count before COVID‐19 (IQR), cells/mm^3^	558 (IQR = 312–719)	–
Baseline HIV VL <50 copies/ml	18	53
cART regimen	PIs (3)	8.8
	TDF or TAF (12)	35
Comorbidity	Cardiovascular disease (5)	27.8
	Chronic lung disease or asthma (2)	11.1
	Diabetes (2)	11.1
	Obesity (2)	11.1
Course of COVID‐19	Clinically unstable patients with respiratory failure (5)	15
	Stable patients with respiratory and/or systemic symptoms (14)	41
	Mild disease without hospitalization (11)	32
	Asymptomatic course (4)	12
COVID‐19 outcome	Full recovery (31)	91
	Death (2)	6

In the study of 34 cases of PLWH diagnosed with COVID‐19, the majority of HIV‐positive patients having full clinical recovery (91%) and COVID‐19 presented mostly as mild disease. Respiratory symptoms were reported in 27 (79.4%) patients while 26 (76.5%) cases presented general symptoms (muscle aches, fatigue/malaise fever); gastrointestinal symptoms (diarrhea, abdominal pain nausea/vomiting) occurred in ten (29.4%) subjects.

Cough was the most common symptom of COVID‐19 and was reported in 24 (70.6%) cases. Malaise/fatigue occurred in 24 (70.6%) and fever in 21 (61.8%) subjects. Muscle aches were observed in 17 (50%) patients, loss of smell in 9 (26.5%) cases, headache in 9 (26.5%) cases, and loss of taste in 7 (20.6%) subjects.

The small group of patients is the main limitation of our study. In our review, we did not report any alarming signals of increased morbidity or mortality from COVID‐19 among HIV‐positive persons among 12 countries in Central and Eastern Europe region.

## AUTHOR CONTRIBUTIONS

All coauthors contributed equally to this work.

## CONFLICT OF INTEREST

All co‐authors declare no conflict of interest.

## CONSENT FOR PUBLICATION

All coauthors consent for this publication.

## PATIENT CONSENT STATEMENT

The Bioethical Committee of the Medical University of Warsaw approved the study (Nr AKBE/155/2020).

## Data Availability

The data that support the findings of this study are available from the corresponding author upon reasonable request.
